# Laser-resistance sensitivity to substrate pit size of multilayer coatings

**DOI:** 10.1038/srep27076

**Published:** 2016-06-01

**Authors:** Yingjie Chai, Meiping Zhu, Hu Wang, Huanbin Xing, Yun Cui, Jian Sun, Kui Yi, Jianda Shao

**Affiliations:** 1 Key Laboratory of Materials for High Power Laser, Shanghai Institute of Optics and Fine Mechanics, Chinese Academy of Sciences, Shanghai 201800, China; 2 University of Chinese Academy of Sciences, Beijing 100049, China

## Abstract

Nanosecond laser-resistance to dielectric multilayer coatings on substrate pits was examined with respect to the electric-field (E-field) enhancement and mechanical properties. The laser-induced damage sensitivity to the shape of the substrate pits has not been directly investigated through experiments, thus preventing clear understanding of the damage mechanism of substrate pits. We performed a systematic and comparative study to reveal the effects of the E-field distributions and localized stress concentration on the damage behaviour of coatings on substrates with pits. To obtain reliable results, substrate pits with different geometries were fabricated using a 520-nm femtosecond laser-processing platform. By using the finite element method, the E-field distribution and localized stress of the pitted region were well simulated. The 1064-nm damage morphologies of the coated pit were directly compared with simulated E-field intensity profiles and stress distributions. To enable further understanding, a simplified geometrical model was established, and the damage mechanism was introduced.

Multilayer dielectric coatings are fluence-limited by rare failures induced by nanosecond laser irradiation^1^,^2^, thus limiting the quality of current low-defect density mirrors. The damage mechanisms of these dielectric multilayers have been studied extensively to maximize the laser-induced damaged threshold (LIDT)^3^. Structural defects, such as scratches^4^, impurities^5^,^6^,^7^,^8^,^9^, and pits^10^ on the substrate, are considered to be among the most important factors limiting the coating function and lifetime. Deformation in the film structure resulting from high or low points on the substrate surface (particles^11^,^12^/artificial nodule seeds^13^,^14^,^15^ or scratches^4^/pits^6^,^10^) yield enhancements in the electric-field (E-field) intensity and therefore reduce the LIDT. Because the multilayer dielectric coating growth exhibits a self-shadowing nature by rotating the substrate during electron-beam (EB) evaporation, the particle defects become nodules that are eventually laid on the surface^14^; however, the pits/scratches are usually buried under the multilayers and cannot be easily observed^10^. However, the pit defects have a relatively low density and random geometrical dimensions, making quantitative research difficult^4^,^10^. Considering the increasing need for large optics with high-power laser-damage resistance, it is very time-consuming and challenging to obtain representative damage morphologies of multilayer coatings on a pitted substrate and to make meaningful comparisons with simulation results.

Recently, the femtosecond-laser fabrication method was applied for creating much smaller pits on a fused silica substrate to prevent the emergence of subsurface cracks that may be induced during the cold-machining process. Microscale pits were ascertained to be one of the laser-damage sources on HR multilayer coatings that cannot be ignored. Femtosecond-laser fabricated pits (fs-pits), whose size can be well controlled, have been used to study the damage behaviours of coatings on pits reliably and efficiently. Unfortunately, studies that used pits of different sizes considered neither the sensitivity of the pit size on the laser-damage resistance nor the mechanical response of deformational multilayer coatings.

In this study, we mainly focused on laser-induced damage on HfO_2_/SiO_2_ multilayer coatings influenced by fs-pits on substrates. A 520-nm femtosecond laser was used for fabricating the micro-pits on the substrates, and a 1064-nm nanosecond laser was used for laser-resistance tests. The LIDT results present a brand-new phenomenon. The coated pit showed different laser-induced damage sensitivities as the pit size changed. Their damage morphologies indicated the damage process and mechanism. simulations were designed to investigate the influence of the E-field distributions and the localized stress on the damage behaviour of the coatings on the pits. Our results show that the pit size affects the damage behaviour of HR coatings.

## Samples and Experiment

### Preparation of fs-pits

All experiments were conducted on the same group of supersonic cleaned fused silica substrates, which were 50 mm in diameter and 5 mm thick. Microscale pits were precisely fabricated by a femtosecond laser platform, details of which are reported elsewhere^16^,^17^. A Ti:sapphire laser system with an operating wavelength of 520 nm, pulse width of 340 fs, and repetition rate of 1 kHz was used. A sweeping mesh comprising femtosecond laser pits with a mesh space of 300 μm was fabricated on a fused silica substrate for the alignment of the laser irradiation during the LIDT test. Before the femtosecond laser was focused on the sample surface by a 10 × OLYMPUS microscope objective, the levelling substrate was fixed on a computer controlled X-Y-Z stage and the laser power was set at 4.6–14.8 mW. The fabricated fs-pits were 2.5–9 μm in diameter and 0.2–0.6 μm deep.

### Preparation of HR coating

EB evaporation was primarily used for depositing the HR coatings because of its potential to achieve the highest LIDT and its wide application in high-power laser facilities^18^. Thus, HR coatings prepared via EB evaporation were investigated in our experiment. HfO_2_ and SiO_2_ were chosen as the high (H)- and low (L)-refractive index materials, respectively. HR coatings with a layered structure of *Substrate/4L(HL)*^*12*^*H4L/Air* were deposited on substrates with and without pits. The HfO_2_ layers were deposited from hafnium metal instead of hafnium oxide to reduce the defect density^19^. SiO_2_ was selected as a low-refractive index material because of its low-loss property^20^. The ambient pressure for both HfO_2_ and SiO_2_ was 2 × 10^−2^ Pa. SiO_2_ was deposited at a rate of ~0.3 nm/s, and HfO_2_ was evaporated at a lower rate of ~0.09 nm/s. Both had a quarter-wave optical thickness at the reference wavelength of 1064 nm (H: 138 nm, L: 183 nm). The refractive indexes of HfO_2_ and SiO_2_ at 1064 nm are 1.912 and 1.449, respectively. The total physical thickness of the film was 5440 nm. All samples had a reflectance higher than 99.5% at 1064 nm. An atomic force microscope was employed to characterize the surface profile morphology of the sample surface before and after coating. The deformational coatings were also characterized by a focused ion beam-scanning electron microscope (FIB-SEM, Carl Zeiss AURIGA Cross Beam) operating at an acceleration voltage of 1 kV.

### Laser-induced damage performance test

The LIDT measurement was implemented in accordance with ISO 21254-1 for 1-on-1 irradiation^21^. A Nd:YAG laser with a 12-ns pulse was operated at a wavelength of 1064 nm. The e^−2^ spot diameters along the X and Y axes were both 416 μm. The damage process was evaluated by comparing the test area before and after the laser irradiation. The X-Y sample stage was adjusted in an attempt to localize every shot to the pitted position^6^,^10^. The site spacing was 1.5 mm, which was 5 times the meshed line space and ~3 times the laser spot diameter, for precise laser shot aiming and preventing any influence from neighbouring damage. Twenty sites were tested for each energy density, and the morphologies of the damaged sites were recorded. The LIDT was defined as the energy density of the incident pulse when the damage probability was 0%. The damage morphologies were characterized by a FIB-SEM.

### Finite-element method (FEM) simulation

The measured LIDT of deformational coatings has been related to the E-field distribution^10^,^14^. Thus, we investigated the dependence of the E-field enhancement on the pit size. The E-field distributions and localized stress on the pitted sites were simulated using FEM. The simulation domain was rectangular and 2D. For the E-field simulation, to obtain accurate results, the rectangular simulation domain was gridded with sufficiently small spaces to ensure that there were at least 10 meshes per wavelength (1064 nm). Furthermore, periodic boundary conditions were applied in the x-direction, and perfectly matched layer boundary conditions were applied in the y-direction. To reduce the back-reflections from the PBCs, a simulation domain with a width of ~70 μm was used for the coating structure, initiating from the maximum pit width of ~7 μm. The simulation was performed using a 1064-nm plane wave as the normal incident field.

The mechanical response of deformational coatings should be considered as a significant factor when damage occurs. For the localized stress simulation, an infinite boundary condition was applied in the x-direction. The relevant mechanical parameters (i.e., Young’s modulus, Poisson ratio) of the multilayer coatings were measured using a nano-indenter (Nano Indenter G200, Agilent) after the EB evaporation. To simulate the local stress in the HR coating, two conditions were utilized: the multilayer structure was bonded into the bulk, and the interface between HfO_2_ and SiO_2_ was ignored; the coefficient of thermal expansion for SiO_2_ and HfO_2_ multilayer coatings was the mean value of 3.45 × 10^−6^ K^−1^ and assumed as a constant value; the same temperature change was applied for the localized stress simulation.

## Results and Discussion

### Geometric modelling of coated substrate pit

**Figure 1:**
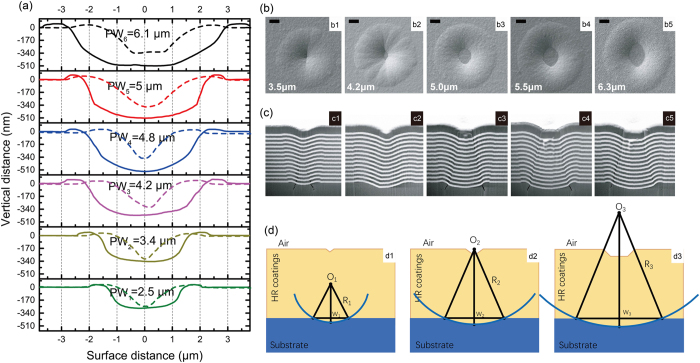
(**a**) Surface profiles of different-sized pits before (dashed lines) and after (solid lines) coating. (**b**) Surface morphologies of the coating for pit sizes of 3.5, 4.2, 5.0, 5.5, and 6.3 μm (b1–b5). PW: Pits width. (**c**) Cross-sections of the pit revealed by FIB technology for corresponding pit sizes (c1–c5) at a magnification of 10,000x. The white layers represent HfO2, and the dark layers represent SiO2. The scale represents 1 μm. (**d**) Schematic of a simple geometric model of a coated substrate pit. W is the radius of the pit, R is the radius of the spherical shell in our hypothesis, and O is the geometric centre of the spherical shell.

As shown in Fig. 1, cross-sectional curves extracted from AFM and FIB-SEM micrographs of the prepared substrate pit were previously examined to reveal the pit geometry. FIB-SEM observations apparently reveal an upside-down nodule. However, compared with the nodule structure on the multilayer coatings, the actual pit defects were not clearly blocked by the particle boundaries. We consider that the shadowing effects of the pits were not as influential as the projecting structure of the nodule^22^,^23^. By fast planetary rotating of the substrate during EB deposition, the difference in the deposition dynamic effects on the fs-pit with a large breadth–to-depth ratio could be ignored. HfO_2_ and SiO_2_ multilayers growth were synchronously perpendicular to both the substrate surface and pit site. For describing the geometric configuration, we developed a simplified model. The first condition utilized was that the pit was regarded as having a shallow spherical shell structure and deformational layers were coated on the substrate pit because of its self-shadowing nature. The second condition was that the voids along the boundaries were neglected. These results showed that the substrate pits prepared by femtosecond laser processing could be equivalent to a spherical shell structure and had an aspect ratio of 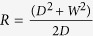, which was obtained by a simple calculation using the Pythagorean Theorem, as shown in Fig. 1(d), where R is the radius of the spherical shell, L is the depth of the pit, and W is the radius of the pit. Notably, this geometrical model leads to the condition that the thickness of a film growing radially outward from a substrate pit is uniform and equal to the thickness of a vertical layer film growing on a perfect substrate, especially along the central axis of the pit. For example, with a coating thickness of 5440 nm, pit-size of 2W = 3.4 μm and D = 330 nm, and a ratio of 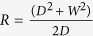, we obtained R-D = 4.21 μm, which means that the centre of the spherical shell was inside the coating and far from the coating surface, as demonstrated in Fig. 1(d1). With a pit-size of 2W = 5.0 μm and D = 512 nm, we obtained R-D = 5.84 μm, which means the shell geometric centre was near the coating surface, as demonstrated by Fig. 1(d2). When the pit width was larger than 5.0 μm, the geometrical centre moved far from the coating surface, as demonstrated in Fig. 1(d3).

### Incident angular range (IAR) analysis

**Figure 2:**
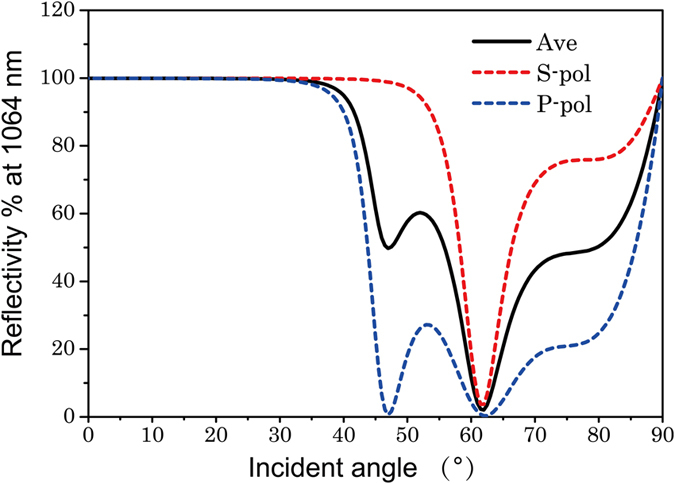
ARB (angular reflectance bandwidth) of the HfO_2_/SiO_2_ HR coatings. The black, red, and blue lines indicate the average polarization, S-polarization, and P-polarization, respectively. The reflection bandwidth was approximately ± 46° for average/S-polarization and ± 32° for P-polarization.

Because the HfO_2_/SiO_2_ HR coatings were irradiated at a normal incidence, the deformational coatings were exposed to a range of incident angles and to both S- and P-polarization at the orthogonal cross-sections. When the point of incidence moved from the pit edge to the centre, the angle of incidence gradually increased from zero to its maximum value. The practical IAR of the deformational coating surface was extracted from the AFM surface profiles of different pit sizes; a comparison is given in Table 1. Interestingly, for pit sizes less than 5 μm, the actual IARs hardly changed. Figure 2 shows that the angular reflection bandwidth (ARB) of the HfO_2_/SiO_2_ HR coatings was limited—approximately ±46° and ±32° for S- and P-polarization, respectively. Consequently, the IAR of the coated pits were smaller than the ARB of the HfO_2_/SiO_2_ HR coatings; thus, the incident laser beam did not penetrate the multilayer stack through the pit centre. The multilayer coatings remained highly reflective even though the coating deformation was induced by the pit with a large breadth-to-depth ratio. Profiles of the coating surface obtained by AFM and the cross section of the multilayer both show that the deformational coatings were not “healed up”, when the pit width was larger than 5.0 μm, with a minor flat round bottom.

### Laser-damage performance of coatings on different-sized pits

**Figure 3:**
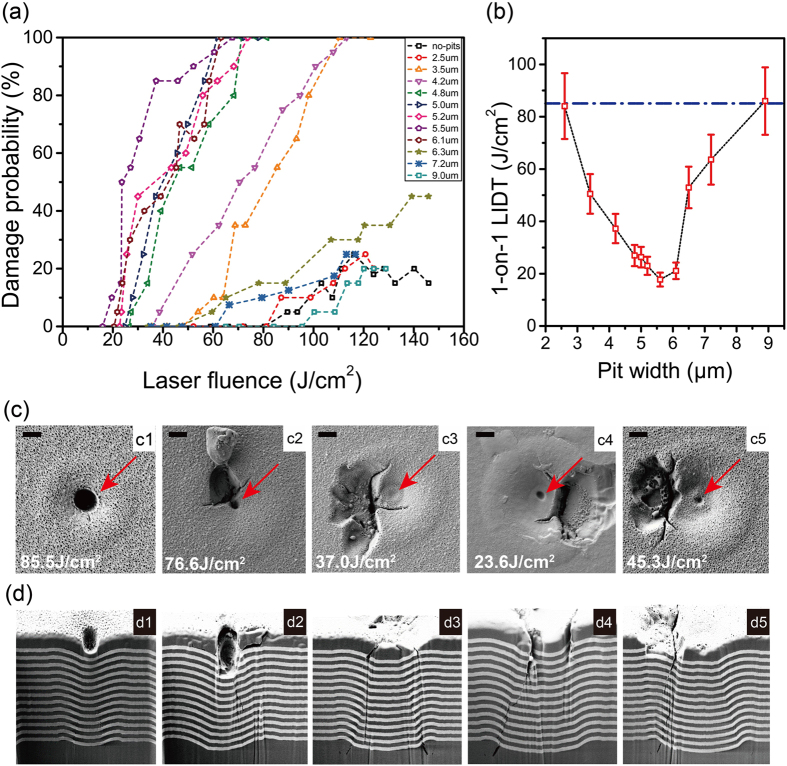
(**a**) 1-on-1 LIDT testing results for coated substrate pit sizes in the range of 2.5–9.0 μm. (**b**) 1-on-1 0% damage probability threshold of deformational HR coatings. The blue dash dot line illustrates the LIDT of coating without pit of 85 J/cm2. (**c**) Damage morphologies for pit sizes of 3.5, 4.2, 5.0, 5.5, and 6.3 μm (c1–c5). The nanoscale melting pit in the centre of the groove bottom is indicated by red arrows (d****) Cross-sections of the damage site revealed by FIB technology (d1–d5) with a magnification of 10,000x. The scale represents 1 μm.

As shown in Fig. 3(a,b), the LIDT results indicate that the laser-damage resistance was clearly influenced by the substrate pit size. The LIDT of the conventional coating (no pits) was higher than 85 J/cm^2^. The LIDT of coating on pits showed a rock bottom when the pit width around 5.4 μm, which was regarded as the most vulnerable deformational structure. Damage to the HR coatings on the pit was observed with a lower fluence. The morphologies of the plasma scald whose source is definitely located in the position of coated pit. As shown in Fig. 3(c), under irradiation with a lower fluence, the groove bottom of the coating surface melted, largely because of the temperature rising. Consequently, a new nanoscale meltdown pit appeared on the centre of the pitted site, as indicated by red arrows in Fig. 3(c). When the coated pit was irradiated with a higher laser fluence, thermomechanical damage occurred around the central melting position. The surface damage morphologies clearly exhibited a combination of melting and fracture. As indicated by FIB-SEM observations, the crack penetrated into the deeper layers and mechanical damage appeared inside the coatings. This critical situation of the LIDT corresponds to the geometric model shown in Fig. 1(d2), where the geometric centre is in the overcoat.

### FEM simulation of the local E-field distribution

**Figure 4:**
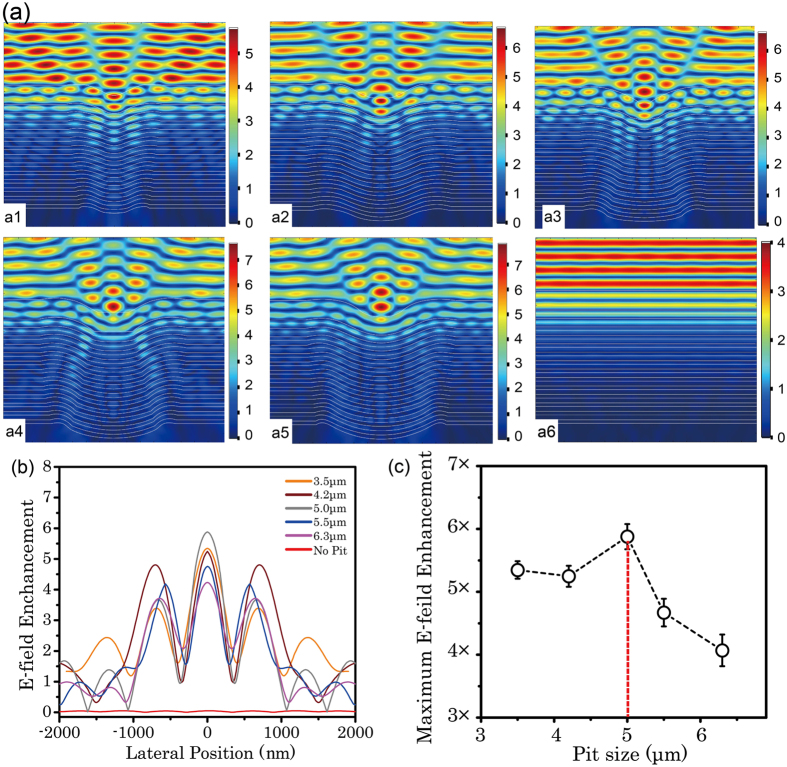
(**a**) Simulated E-field distributions of coated pits width of 3.5, 4.2, 5.0, 5.5, and 6.3 μm (a1-a5) and No-pit (a6), respectively. The white lines represent the coating stacks. The colour scale is different for different pits. (**b**) Simulated E-field distributions along the coating surface for deformational coatings and conventional coatings (No Pit) on different-sized pits. The lateral zero position was aligned to the groove bottom centre of the coated pit. (**c**) Maximum simulated E-field value along the coating surface.

To determine whether the substrate pit can yield E-field intensification within HR coatings, a cross-sectional image of HfO_2_/SiO_2_ was used to estimate the E-field distribution. Figure 4 shows the S-polarized E-field distribution for five kinds of multilayers coated on substrate pits with sizes of 3.5, 4.2, 5.0, 5.5, and 6.3 μm and conventional coatings. The profiles of the reflective index were obtained by processing actual FIB-SEM images, as shown in Fig. 1. The differences in the E-field distribution among the five deformational geometries were drastic, especially with respect to the distribution at the interface between the air and the coatings. The observed difference in the E-field distribution can be quantitatively explained as follows: a light beam-focusing phenomenon was revealed in the axis of the pit because of their HR nature, as demonstrated by the E-field distribution and their value along the axis of deformational coating in Fig. 4(a). Without regard for the E-field in the air, the E-field was the maximum at the groove bottom of the pitted coating, which was just located at the nanoscale melting central pit indicated by red arrows in Fig. 3(c). We extracted the E-field distribution along the curved interface between the air and the coating, and the maximum E-field distribution reached to a peak when the pit width was 5.4 μm, as shown in Fig. 4(b). The maximum value of interface E-field led to the minimum value of LIDT because the LIDT was related to the E-field distribution to a certain extent. Besides, as shown in Fig. 4(b), the E-field enhancement was located on the deformational area and the delamination occurred due to the laser energy deposition. The E-field of the interface between the air and the overcoat is usually set as 0 to achieve a high laser-damage resistance. However, the simulated results show that light intensification by a factor as large as 4–6 can occur in the air/coating interface, as shown in Fig. 4(c). The deformational coating was responsible for the E-field deformation and the maximum E-field movement, which eventually caused surface damage.

### Localized stress distribution according to FEM simulation

**Figure 5:**
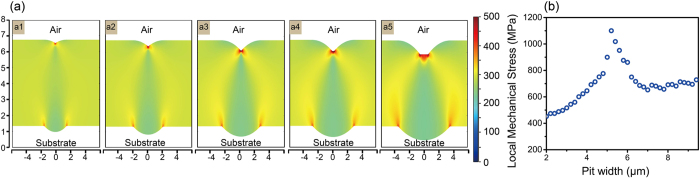
(**a**) Localized mechanical stress distribution inside the deformational coating on pits with sizes of 3.5, 4.2, 5.0, 5.5, and 6.3 μm (a1–a5, respectively), with the same temperature increase of 1600 K. The simulated geometric model from the R = (D2+W2)/2D geometry was used. (**b**) Maximum stress extracted from the FEM mechanical simulation results.

The E-field simulation results explain the origin of the damage. However, the light intensification of 4–6 in the coating/air interface was not so high as to result in damage threshold ranges from lower than 20 J/cm^2^ to higher than 80 J/cm^2^. The mechanical properties must be considered a significant factor in the nanosecond laser-damage region. After being irradiated by a high-power laser pulse, the coating becomes extremely hot in a very short time, yielding thermal meltdowns on the surface and mechanical stress release in the coatings. The localized mechanical stress caused by a temperature increase can be calculated by using a linear elastic material model for a nearly incompressible material^24^. In the FEM simulation, we used the temperature increase of ΔT = 1600 K in order to calculate the mechanical stress distribution in the coating^10^. As shown in Fig. 5, the simulation results indicate the concentration of the stress in the deformational coating. The mechanical stress concentration on the groove bottom was responsible for the delamination on surface. As showed in Fig. 3(d), a fracture emerged along the radial direction of the deformational section because of the mismatch in the mechanical stress during temperature increase, as shown in Fig. 5(a). After extraction of the maximum mechanical stress, a turning point appeared at D = 5.2 μm, which was the most vulnerable coating structure. For different ΔT values, further investigations showed the vulnerable pit size was only influenced by the geometric structure of the coatings. After comparing this result with the LIDT result, we believe that it is not a coincidence that the laser-damage resistance was minimized when the mechanical stress was maximized. According to the geometric model, when the shell centre is closer to the coating surface, the mechanical properties of the deformational coating are poorer.

### Sensitivity analysis of LIDT

In this experiment, the negative impact of substrate pit on the laser damage resistance of multilayer coatings was confirmed. Different-sized pits showed different sensitivities on laser-resistance, and a pit width of around 5 μm was easily damaged when irradiated by lower laser fluence, because both the E-field and localized mechanical stress showed a turning point. At the turning point, the maximum E-field enhancement in the groove bottom of the coated pit led to significant surface melting and delamination, and the maximum localized mechanical stress led to serious internal crack failure. Similarly, in the geometric model, the laser-resistance performance of coating on pits was low when the shell centre was closer to the coating surface.

## Conclusion

We attempted to find a link between the E-field distributions, localized mechanical stress, and damage morphologies of coatings on pitted substrates and demonstrate exactly how the E-field distributions and local stress affect the thermomechanical damage on deformational coatings. However, the IAR of the coated pits were smaller than the ARB of the HfO_2_/SiO_2_ HR coatings, which indicated the incident laser beam did not penetrate the multilayer stack through the pit centre. The light intensification in the surface of the overcoat resulted in melting of the groove bottom. The melted regions led to considerable thermal pressure, eventually causing delamination of the deformational overcoat around the melted regions, leaving a nanoscale pit in the spot centre and subsequent damage around it. Then, the delaminated material from the melting region induced a plasma explosion on the coating surface, leaving a large-scale scald with temperature increase. Finally, the temperature change yielded a localized mechanical stress concentration and an internal fracture below the coating surface. The E-field simulation and localized stress simulation clearly indicate that the pit on the substrate significantly influenced both the E-field distribution at the air/coating interface and the stress concentration in the deformational coating. We believe that the E-field distribution as well as the local mechanical properties significantly affects the damage behaviour of the coating on a pitted substrate, and the damage morphologies can reflect both these causes. Considering the relationship between the geometric change of the multilayers and the laser-damage performance, a very simple empirical law was proposed according to our experiment. By using an aspect ratio of 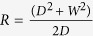, the dangerous pit size for a specific coating design can be estimated and corresponding measures could be taken for preventing the formation of a vulnerable coating structure. For example, flattening the pit by localized polishing, or using a thick SiO_2_ undercoat as a suture layer could be considered.

## Additional Information

**How to cite this article**: Chai, Y. *et al*. Laser-resistance sensitivity to substrate pit size of multilayer coatings. *Sci. Rep.* **6**, 27076; doi: 10.1038/srep27076 (2016).

## Figures and Tables

**Table 1:** IAR of deformational coatings extracted from the practical AFM image.

**Pit width**	7.2	6.1	5.0	4.8	4.2	3.4	2.5
**IAR (°)**	±26	±24	±20	±17	±17	±17	±17
